# Getting It Straight: Accommodating Rectilinear Behavior in Captive Snakes—A Review of Recommendations and Their Evidence Base

**DOI:** 10.3390/ani11051459

**Published:** 2021-05-19

**Authors:** Clifford Warwick, Rachel Grant, Catrina Steedman, Tiffani J. Howell, Phillip C. Arena, Angelo J. L. Lambiris, Ann-Elizabeth Nash, Mike Jessop, Anthony Pilny, Melissa Amarello, Steve Gorzula, Marisa Spain, Adrian Walton, Emma Nicholas, Karen Mancera, Martin Whitehead, Albert Martínez-Silvestre, Vanessa Cadenas, Alexandra Whittaker, Alix Wilson

**Affiliations:** 1 Emergent Disease Foundation, Suite 114, 80 Churchill Square Business Centre, King’s Hill, Kent ME19 4YU, UK; catrinasteedman@gmail.com (C.S.); angeloselukwe@gmail.com (A.J.L.L.); 2 School of Applied Sciences, London South Bank University, 103 Borough Rd, London SE1 0AA, UK; drrachelgrant@gmail.com; 3 School of Psychology and Public Health, La Trobe University, Bendigo, VIC 3552, Australia; t.howell@latrobe.edu.au; 4 Pro-Vice Chancellor (Education) Department, Murdoch University, Mandurah, WA 6210, Australia; phil@ecoarena.com.au; 5 Colorado Reptile Humane Society, 13941 Elmore Road, Longmont, Colorado, CO 80504, USA; nash@corhs.org; 6 Veterinary Expert, P.O. Box 575, Swansea SA8 9AW, UK; mike@jessop.uk.net; 7 Arizona Exotic Animal Hospital, 2340 E Beardsley Road Ste 100, Phoenix, Arizona, AZ 85024, USA; apilny@azeah.com; 8 Advocates for Snake Preservation, P.O. Box 2752, Silver City, NM 88062, USA; mel@snakes.ngo; 9 Freelance Consultant, 7724 Glenister Drive, Springfield, VA 22152, USA; stevegorzula@gmail.com; 10 Jacksonville Zoo and Gardens, 370 Zoo Parkway, Jacksonville, FL 32218, USA; spainmarisa@outlook.com; 11 Dewdney Animal Hospital, 11965 228th Street, Maple Ridge, BC V2X 6M1, Canada; dewdneyvet@gmail.com; 12 Notting Hill Medivet, 106 Talbot Road, London W11 1JR, UK; Emma.nicholas@medivet.co.uk; 13 Facultad deMedicina Veterinaria y Zootecnia, Universidad Nacional Autónoma de México, Avenida Insurgentes Sur s/n, Ciudad Universitaria CDMX, Ciudad de México 04510, Mexico; dra.kelokumpu@gmail.com; 14 Chipping Norton Veterinary Hospital, Banbury Road, Chipping Norton OX7 5SY, UK; martincnvets@gmail.com; 15 Catalonian Reptiles and Amphibians Rescue Centre (CRARC), 08783 Masquefa, Spain; crarc-masquefa@outlook.com; 16 Animal Protection Biodiversity & Environment Section, Government of Catalonia, 43004 Tarragona, Spain; vanessa.cadenas@gencat.cat; 17 School of Animal and Veterinary Sciences, University of Adelaide, Roseworthy, SA 5371, Australia; alexandra.whittaker@adelaide.edu.au; 18 Center for Avian and Exotic Medicine, 562 Columbus Avenue, New York, NY 10024, USA; awilson@avianandexoticvets.com

**Keywords:** literature review, reptile husbandry, enclosure size, space, body posture

## Abstract

**Simple Summary:**

Snakes are sentient animals and should be subject to the accepted general welfare principles of other species. However, they are also the only vertebrates commonly housed in conditions that prevent them from adopting rectilinear behavior (ability to fully stretch out). We conducted a literature search and review regarding recommendations for enclosure sizes for snakes. We found that recommendations suggesting enclosure sizes shorter than the snakes were based entirely on decades-old ‘rule of thumb’ practices that were unsupported by scientific evidence. In contrast, recommendations suggesting enclosure sizes that allowed snakes to fully stretch (rectilinear posture) utilized scientific evidence and considerations of animal welfare. Rectilinear behavior is normal, distinct, and common across snake species, and is essential and fundamental to snake health and welfare. Scientific evidence-based recommendations for providing enclosures allowing snakes to fully stretch now constitute mainstream guidance information and good practice as a minimum spatial provision, both during short-term and long-term situations.

**Abstract:**

Snakes are sentient animals and should be subject to the accepted general welfare principles of other species. However, they are also the only vertebrates commonly housed in conditions that prevent them from adopting rectilinear behavior (ability to fully stretch out). To assess the evidence bases for historical and current guidance on snake spatial considerations, we conducted a literature search and review regarding recommendations consistent with or specifying ≥1 × and <1 × snake length enclosure size. We identified 65 publications referring to snake enclosure sizes, which were separated into three categories: peer-reviewed literature (article or chapter appearing in a peer-reviewed journal or book, *n* = 31), grey literature (government or other report or scientific letter, *n* = 18), and opaque literature (non-scientifically indexed reports, care sheets, articles, husbandry books, website or other information for which originating source is not based on scientific evidence or where scientific evidence was not provided, *n* = 16). We found that recommendations suggesting enclosure sizes shorter than the snakes were based entirely on decades-old ‘rule of thumb’ practices that were unsupported by scientific evidence. In contrast, recommendations suggesting enclosure sizes that allowed snakes to fully stretch utilized scientific evidence and considerations of animal welfare. Providing snakes with enclosures that enable them to fully stretch does not suggest that so doing allows adequate space for all necessary normal and important considerations. However, such enclosures are vital to allow for a limited number of essential welfare-associated behaviors, of which rectilinear posturing is one, making them absolute minimum facilities even for short-term housing.

## 1. Introduction

Snakes are kept in captivity in a variety of situations, including zoo exhibits; laboratories; culinary, skin, and curio producers; various pet industry facilities; and in private homes [1,2,3,4]. Reptiles generally are subject to many misconceptions and underestimations regarding both their lives in nature and their needs in captivity [3,5,6], leading to an existence in captivity of frequent deprivation, even in the best zoo facilities [7]. Implicit bias—much of it embedded in popular culture—can also be identified as a source of distorted perceptions that may mislead researchers and the public alike [3,5]. One perennial issue is that many snakes are confined to enclosures in which they cannot fully stretch their bodies, notably in the exotic pet trading and keeping sector [8]. For example, a recent survey [9] found that 42% of private snake keepers held their animals in vivaria where they could not stretch out.

Reptiles, including snakes, are increasingly recognized for their behavioral and cognitive complexities, as well as for their physiological and anatomical mechanisms for processing pain and stress [7,10,11,12,13,14,15,16]. Snakes are sentient animals with relevant attributes comparable to avian and mammalian taxa [17,18]; they all share an ancestral heritage suggesting such sentience is present within all these animal groups. Accordingly, these reptiles should be considered subject to the accepted sensitivities and general welfare principles currently afforded other species.

### 1.1. Established Animal Welfare Principles

Principles common to animal welfare science include encouraging positive welfare states and discouraging negative welfare states. Welfare states are characterized by an animal’s physical condition as well as by its subjective mental state, and—perhaps fundamentally—the extent to which an animal may manifest control or ‘individual agency’ over interactions with its environment [19,20,21,22]. For example, Broom [21] described five factors illustrating where control, or lack of it, may impact animal welfare (Table 1).

These welfare factors have been used to develop enduring guidelines, such as the Five Freedoms [23,24], and the more recent Five Domains Model [25] (Table 2), that apply to all animals.

In essence, the Five Domains Model uses several elements that seek to identify establishment of positive states, rather than merely avoidance of negative states, and where successful, these positive states should culminate in a positive mental state—or a defining indication of a ‘life worth living’. As indicated using bold type in Table 2, each of those factors as a minimum are relevant to promoting positive or (if unmet) negative welfare states in snakes [8,26].

### 1.2. Established Welfare Principles Applied to Snakes

Although several welfare models (as above) are important, we have focused on the relevance of the five points developed by Broom [21], and we considered these specifically in relation to snakes. Environments that do not permit snakes to fully stretch their bodies are clearly contrary to welfare principle 1. Snakes that cannot fully stretch (or express other spatially dependent behaviors) or escape confinement are compromised under welfare principle 2. Unenriched and spatially deprived enclosures do not allow for welfare principle 3. Snakes, like all reptiles, have strong ancestral (innate) traits and associated drive states, including locomotor and exploratory behaviors that are thwarted under spatially restrictive conditions, thus compromising welfare principle 4. Extraneous disturbances due to handling, noise, light, conspecific, or other disturbances are probably relevant stimuli compromising welfare principle 5.

In addition, there are numerous reptile- and snake-specific welfare considerations. Fundamental ectothermy and its associated precise thermoregulatory needs are highly relevant to physiological, behavioral, and psychological states [10,15,16,27]. Very small enclosures limit thermal (and light—including UVB) gradients, make humidity and temperature control difficult, potentially affecting holistic health. Spatial deficiencies also restrict exercise and reduce the amount of enrichment that can be provided, in the form of physical ‘cage furniture’, such as hides, pools, and branches, and in the form of environmental variation of temperature and lighting.

In snakes, strong innate ancestral traits greatly determine ontogenetic behavioral and psychological states [7,11,28]. Particular metabolic and energetic dynamics affect behavioral responses, immunity, and healing [10,29]. Nocturnalism, which is common among snakes, typically conflicts with human activity patterns, invites disturbances, and compromises welfare assessments by observers [8,27].

### 1.3. Snake Rectilinear Behavior and Ability to Fully Stretch

Snake rectilinear behavior (i.e., adopting a straight-line or near straight-line posture—stretching-out) is a common, distinct, and normal feature of snake biology during locomotion and rest activities [8,30]. Several studies have examined snake rectilinear behavior in captivity, and its importance. For example, a study of rectilinear behavior among 65 snakes of 31 species at eight zoos in Canada and the UK and found that within one hour of observation 24 snakes (37%) of 14 species (45%) ‘stretched-out’ [8]. Given that this study was conducted under diurnal conditions, whereas many or most snakes are nocturnal, crepuscular, or fossorial, the results may, in fact, underestimate rectilinear behavior in snakes, and confirm that adoption of rectilinear positions is a common and normal component of snake behavior among individuals generally and across a range of species. That investigation also documented a range of 22 reported clinical problems (e.g., rostral abrasions, dermatitis, obesity, infection, co-occupant injury, constipation, and degenerative joint disease) and 24 behavioral problems (e.g., interaction with transparent boundaries, hyperactivity, hypoactivity, co-occupant aggression, hyperalertness, head-hiding, and freezing) associated with confinement of snakes in smaller enclosures, with an emphasis on housing that prevented rectilinear behavior. The findings for this study [8] were supported by expert opinion from veterinarians specializing in exotic species.

Another study [26] showed that snakes in smaller and/or less enriched enclosures, notably those in which they could not fully stretch, displayed greater signs of stress than those in larger more enriched enclosures, and that important normal behaviors are thwarted under more restrictive conditions, concluding that snakes must be able to perform rectilinear behavior within enclosures. For example, observations of positive and negative behavioral welfare indicators among 35 captive-bred ball pythons (*Python regius*) compared larger enriched enclosures in which snakes could fully stretch their bodies with those kept in rack system housing, which is well-known to involve minimalistic and spatially highly restrictive conditions [26].

Results showed that snakes in racks experienced considerable restriction regarding species-typical behaviors, and the study concluded that such housing did not meet acceptable welfare standards. Another study involving over 700 snakes in private homes showed that in vivaria <1 × snake length (SL) there were more clinical signs of stress than those in >1 × SL conditions; thus, snakes in smaller enclosures exhibited more signs of captivity-stress [31]. A list of clinical signs of stress can be found in Warwick et al. [8], examples of which are cited above.

Numerous other experimental and review studies show that snakes display preferences for, and greater security in, larger more naturalistic conditions, and that such environments favor snake welfare (e.g., [28,32,33,34,35,36,37,38,39,40,41]). Environments that do not address normal biological and behavioral needs (e.g., regarding space, thermal, lighting, and humidity ranges, interactive enrichment provisions, and social elements (where relevant)), should be considered incongruent with snake welfare (‘negative states’). Relatedly, factors consistent with snake welfare (‘positive states’) [25] are also important, as provided in Mellor’s Five Domains model (Table 2).

Rectilinear movements and postures are considered to be associated with expression and facilitation of comfort (e.g., relaxed state and muscular health), and the relief of discomfort (e.g., digestive tensions) in snakes (e.g., [8,26,28,39]). Accordingly, ability to perform rectilinear behavior is relevant both in the context of avoiding negative states (i.e., its deprivation leads to stress and harm) as well as achieving positive states (i.e., expression of quiescence and comfort). Although snakes may exhibit one or more minor body curvatures during some rectilinear movements or postures, such behavior does not detract from the importance of accommodating the full-length of the animals. Overly small and restrictive enclosures inherently also limit the inclusion of habitat features that may promote normal and natural movement, thus reducing behaviors that can be expressed. It is important that guidelines for snake husbandry use the best, most recent recommendations based on the weight of available evidence. Despite this, much guidance, both historical and present, advocates insufficient space to meet the needs of captive snakes—which are the only vertebrates regularly prevented from being able to extend to their full length in their cages, or vivaria. Therefore, in this article we review and summarize the literature, and the available evidence, for and against the need to allow captive snakes to stretch out fully in their enclosures.

## 2. Materials and Methods

Review literature was provided from authors’ libraries and supplemented with a systematic Google Scholar (unlimited time frame) search, thus filtering out non-scientific materials, and Google searches for peer-reviewed publications using the following terms: ‘snake’ + ‘accommodation’ + ‘space’ + ‘spatial’ + ‘vivarium’ + ‘enclosure’ + ‘cage’ + ‘length’, as progressive new search additions. Other published materials relating to snake spatial considerations that originated in veterinary journals, position statements, and relevant reports were also included where identified, although separately from the tabulated summary of peer-reviewed literature.

We separated literature into three categories (peer-reviewed literature, grey literature, and opaque literature) according to their following characteristics: peer-reviewed literature = article or chapter appearing in a peer-reviewed journal or book; grey literature = government or other report or scientific letter identified through Google Scholar; opaque literature = non-scientifically indexed reports, care sheets, articles, husbandry books, websites or other information for which originating source was not based on scientific evidence or where scientific evidence was not provided.

**Figure 1:**
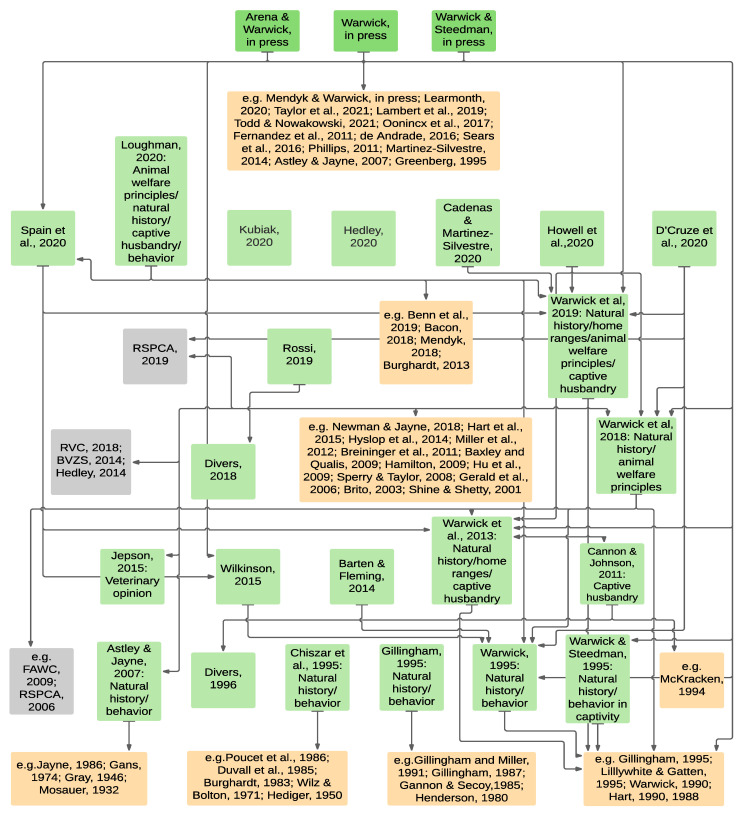
Peer-reviewed evidence-base showing primary cross-referencing of sources for recommended minimum snake enclosure dimensions derived from Table 3 and consistent with ≥1 × SL. Arrows direct to previously published material. Green boxes = peer-reviewed citations in Table 3 that specifically relate to enclosure sizes for snakes. Amber boxes = citations of general biological or behavioral nature referenced as supportive information. Grey boxes = citations of grey literature sources referenced as supportive information. Grey boxes indicate non-peer-reviewed (grey literature) publications with recommendations. Cited references in Figure 1: Arena and Warwick, in press [39]; Mendyk and Warwick, in press [5]; Warwick, in press [27]; Warwick and Steedman, in press [40]; Taylor et al., 2021 [59]; Todd and Nowakowski et al., 2021 [60]; Hedley, 2020 [53]; Kubiak, 2020 [54]; Cadenas and Martinez- Silvestre, 2020 [36]; D’Cruze et al., 2020 [52]; Howell et al., 2020 [9]; Learmonth, 2020 [18]; Loughman, 2020 [37]; Spain et al., 2020 [38]; Benn et al., 2019 [61]; Lambert et al., 2019 [17]; RSPCA, 2019 [62,63,64,65]; Warwick et al, 2019 [8]; Bacon, 2018 [66]; Divers, 2018 [49]; Mendyk, 2018 [3]; Rossi, 2018 [50]; RVC, 2018 [67,68]; Warwick et al, 2018 [51]; Newman and Jayne, 2018 [69]; Oonincx et al., 2017 [70]; de Andrade, 2016 [71]; Sears et al., 2016 [72]; Hart et al., 2015 [73]; Jepson, 2015 [47]; Wilkinson, 2015 [48]; Barten and Fleming, 2014 [46]; BVZS, 2014 [74]; Hedley, 2014 [75]; Hyslop et al., 2014 [76]; Martinez-Silvestre, 2014 [77]; Burghardt, 2013 [7]; Warwick et al., 2013 [34]; Miller et al., 2012 [78]; Cannon and Johnson, 2011 [45]; Phillips, 2011 [79]; Breininger et al., 2011 [80]; Fernandez et al., 2011 [81]; Baxley and Qualis, 2009 [82]; FAWC, 2009 [24]; Hamilton, 2009 [83]; Hu et al., 2009 [84]; Sperry and Taylor, 2008 [85]; Astley and Jayne, 2007 [44]; Gerald et al., 2006 [86]; RSPCA 2006 [87]; Brito, 2003 [88]; Shine and Shetty, 2001 [89]; Divers, 1996 [43]; Chiszar et al., 1995 [42]; Gillingham, 1995 [11]; Greenberg, 1995 [90]; Lillywhite and Gatten Jr., 1995 [10]; Warwick, 1995 [28]; Warwick and Steedman, 1995 [32]; McKracken, 1994 [91]; Gillingham and Miller, 1991 [92]; Hart, 1990 [93], 1988 [94]; Warwick, 1990 [95]; Gillingham, 1987 [96]; Jayne, 1986 [97]; Poucet et al., 1986 [98]; Duvall et al., 1985 [99]; Gannon and Secoy, 1985 [100]; Burghardt, 1983 [101]; Henderson, 1980 [102]; Gans, 1974 [103]; Wilz and Bolton, 1971 [104]; Hediger, 1950 [105]; Gray, 1946 [106]; Mosauer, 1932 [107].

Two cited studies [26,31] do not feature in Table 3 or Figure 1 because, at the present time, they remain in the peer-review and publication process. However, because one of these studies [26] has a ‘doi’ reference, we have included it as presently grey literature in Table 5, and we appreciate that this may (along with reference [31]) advance to the full peer-reviewed and published status in due course, in which case they would both qualify for Table 3.

## 3. Results

The Google Scholar literature search identified 31 peer-reviewed and 18 grey literature publications, and a further 16 opaque publications were identified both incidentally and within authors’ libraries (total 65 publications). The search did not identify any publications not already contained in authors’ libraries or located by individual authors.

### 3.1. Peer-Reviewed Literature

Peer-reviewed recommendations consistent with or specifying ≥1 × SL enclosures are provided in Table 3 (25 publications), and those that are not consistent with that recommendation are provided in Table 4 (6 publications).

**Figure 2:**
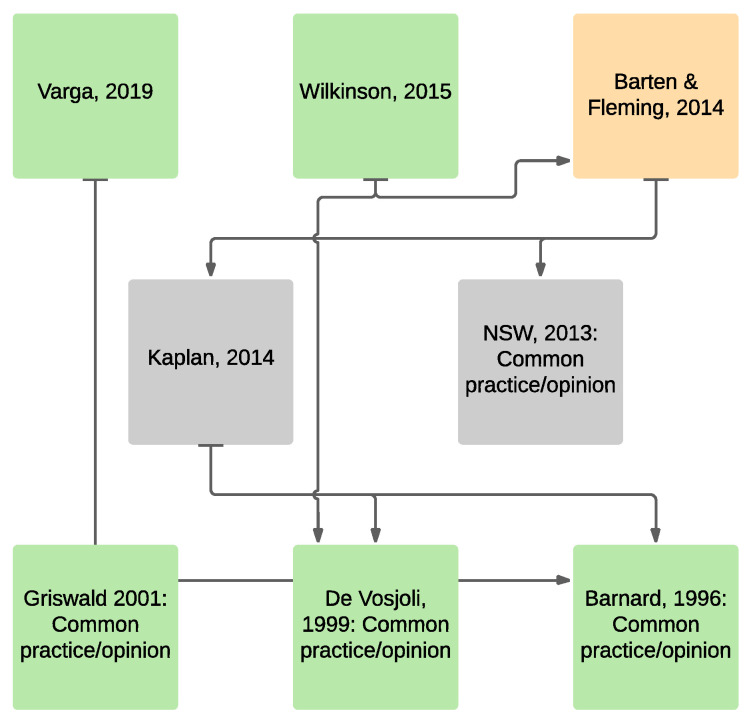
Peer-reviewed evidence-base showing primary cross-referencing of sources for recommended minimum snake enclosure dimensions derived from Table 4 and consistent with <1 × SL. Arrows direct to previously published material. Green boxes = peer-reviewed citations in Table 4 that specifically relate to enclosure sizes for snakes. Amber boxes = citations of general biological or behavioral nature referenced as supportive information. Grey boxes = citations of grey literature sources referenced as supportive information. Cited references in Figure 2: Varga, 2019 [58]; Wilkinson, 2015 [48]; Barten and Fleming, 2014 [46]; Kaplan, 2014 [108]; NSW, 2013 [109]; Griswald 2001 [57]; De Vosjoli, 1999 [56]; Barnard, 1996 [55].

Figure 1 and Figure 2 depict the peer-reviewed evidence-base for minimum snake enclosure dimensions derived from Table 3 and Table 4. Green boxes represent peer-reviewed citations in Table 3 and Table 4 that specifically relate to enclosure sizes for snakes. Amber boxes represent citations of general biological or behavioral nature that are cited as supportive information for recommendations contained in green box publications. Grey boxes represent grey literature sources. Figure 1 illustrates the broad and scientifically robust information bases supporting the literature for ≥1 × SL dimensions. Figure 2 illustrates the narrow and unscientific information bases for literature < 1 × SL dimensions. Some grey literature information is also shown in Figure 1 and Figure 2 where peer-reviewed and grey literature information cross-reference. The grey literature from Table 5 and Table 6 is not depicted in figures, although it is worth noting that the cited grey literature recommending < 1 × SL either has no reference base or cites references that are solely based on common practice or opinion, whereas the majority of the cited grey literature recommending ≥ 1 × SL references peer reviewed publications or objective biological and veterinary opinion.

### 3.2. Grey and Opaque Literature

Author libraries and additional regular Google searches identified numerous examples of literature addressing the issues of snake enclosure size and whether or not to provide space for snakes to fully stretch.

#### 3.2.1. Grey Literature

Table 5 and Table 6 list identified publications (e.g., guidance policy and debate) from the recent grey literature supporting the ≥1 × SL enclosure size recommendation (11 publications), and those not supporting it (7 publications), respectively.

#### 3.2.2. Opaque Literature

We acknowledge that there are many husbandry publications, including printed care sheets and books, as well as online guides that contain some reference to snake enclosure sizes, and it is likely that liberally among these is mention of whether or not snakes should be able to fully stretch. For practical reasons, and because our focus was to address the more substantial literature, it was not relevant to review and include such a plethora of opaque material, and given that we have focused on the primary scientific literature, we did not consider the general omission of those publications to be detrimental to this investigation.

Nevertheless, we noted 16 opaque sources referring to snake enclosure sizes, and of these we included 2 particular sources [120,121] because these books, which are based on common practice and opinion, are regularly cited in snake husbandry recommendations. Banks [120] recommends > 1 × SL and <1 × SL according to species, whereas McCurley [121] recommends 75% SL for ball pythons (*Python regius*). There were also a number of more minor publications including editorials and letters to editors of veterinary journals promoting enclosures consistent with ≥1 × SL [75,114,122,123,124,125,126,127,128,129] versus enclosures consistent with <1 × SL [130,131,132,133].

## 4. Discussion

### 4.1. Nature of Reviewed Evidence and Information

The nature of evidence and information giving rise to the recommendations for certain enclosure sizes or acknowledgement of their importance contained in Table 3, Table 4, Table 5 and Table 6 was highly variable. Below we present and discuss, in particular, key themes regarding the evidence and information in Table 3, Table 4, Table 5 and Table 6.

#### 4.1.1. Peer-Reviewed Literature Consistent with ≥1 × SL

In the peer-reviewed literature in Table 3 (recommending ≥ 1 × SL minimum enclosures), information was substantively based on five identifiable criteria: 1. normal (roaming and rectilinear or near-rectilinear) behavior among wild and captive snakes [8,11,27,28,32,34,39,40,44,46,47,51,53,54]; 2. normal home ranges among wild snakes [8,34,39,40]; 3. reported stress-related behavior, injury or disease among captive snakes, with increasing prevalence among animals in smaller enclosures [8,27,28,34,40,42]; 4. established governing principles for animal welfare science (e.g., control over environment, expression of preference, hard-wired behavioral needs, five freedoms) [8,27,34,36,38,39,40]; and 5. carrying forward recommendations based on 1–4 above, experience, opinion, or avoidance of harm [9,36,43,45,48,49,50,52]. One review [47], and some anecdotal contributions, suggest that enclosures with a diagonal dimension that meet the ≥1 × SL could be acceptable. However, this approach does not account for important environmental furnishings, which ought to be liberally arranged in an enclosure, and also greatly limits optional direction to stretch. Thus, the breadth of the literature base in this section involved research, natural history, behavior, home ranges, common practice, and opinion.

#### 4.1.2. Peer-Reviewed Literature Consistent with <1 × SL

In the peer-reviewed literature in Table 4 (recommending <1 × SL minimum standard), information was substantively based on two identifiable criteria: 1. common practice or ‘rule of thumb’ (term as used by authors of original relevant publications); and 2. carrying forward recommendations or opinion based on 1 above, experience, or opinion [46,48,56,58]. Thus, the literature base in this section involves common practice and opinion.

#### 4.1.3. Grey Literature Consistent with ≥1 × SL

The grey literature consistent with ≥1 × SL is derived from consensus-based position or policy statements by major animal welfare and veterinary representative bodies such as the Royal Society for the Prevention of Cruelty to Animals, the Royal Veterinary College, the British Veterinary Association, and the British Veterinary Zoological Society [62,63,64,65,67,68,112,114], and reports and general articles authored by relevantly qualified impartial scientists and veterinarians [115,116,122]. All of these examples referred back to several peer-reviewed publications [3,8,47,51,134] or to other veterinarian and biologist consensuses based on a knowledge of the natural history of particular species of reptiles. Accordingly, this literature, while not subject to classical peer-review, nevertheless reflected results of high-level objective technical opinions.

#### 4.1.4. Grey Literature Consistent with <1 × SL

The grey literature consistent with <1 × SL was derived from governmental guidance [109,118,119] and from a well-regarded website resource [108]. Governmental guidance characteristically is based on conducting consultation exercises to provide broad consensus information from diverse stakeholders. The Victoria State Government’s [119] basis for their recommendations are two sources [55,120], which provide arbitrary opinion. Of note, the English Government guidance [118] originally included a 1 x SL specification; however, this was deleted at the request of the pet trade and hobby community [123]. The aforementioned website resource [108] carries forward recommendations based on two peer-reviewed items [55,56], both of which are themselves founded on opinion or ‘rule of thumb’.

### 4.2. Literature Review Summary

Based on this review, the original foundations and subsequent common recommendations for <1 × SL enclosures are primarily derived from two non-scientific snake keeper-breeder resources [120,121], and involve a rationale guided entirely by personal opinion and traditional practice. This non-scientific guidance has been frequently repeated, with little challenge to its veracity. Consequently, many non-professional, and at least some professional, reptile keepers use overly restrictive enclosures. Relevantly, we found no scientific studies to show that smaller and unnaturalistic enclosures were not detrimental to the animals. In addition, recent research, advice, and practices showed a paradigm shift towards evidence-based husbandry, which does not support earlier guidance promoting smaller and unnaturalistic snake enclosures. Of the 25 peer-reviewed publications consistent with the ≥1 × SL recommendation, 10 reported results of original research, whereas of the 6 peer-reviewed publications consistent with the <1 × SL recommendation, none reported results of original research.

Although some grey literature sources referred to a peer-reviewed source, such peer-reviewed sources are, in many cases, redirected to grey and opaque literature information. Some peer-reviewed literature regarding snake spatial habits in nature, and husbandry requirements in captivity, includes information that has been carried forward in reviews of original research. Such information has, of course, also been further scrutinized for integrity during the peer-review process prior to publication. In contrast, grey and opaque literature involved no such accountability in terms of accuracy, validity, relevance, and significance.

### 4.3. Drivers of Recommendations

Biologists, veterinarians, and veterinary bodies (e.g., in the UK the British Veterinary Association, British Veterinary Zoological Society, Royal Veterinary College) frequently promote the use of enclosures consistent with ≥1 × SL. This recommendation is based on considerations of natural history, normal and abnormal behavior, established principles of animal welfare science, avoidance of negative clinical and behavioral consequences, obligations to use objective and impartial information, and avoidance of harm (e.g., [8,28,32,34,36,38,44,45,47,50,51,52]). The recommendation is also based on approaches that promote normal behavior, exercise and joint mobilization to, for example, avoid spinal disorders as a part of physical rehabilitation to improve range of motion and neuroproprioceptive training [135].

Animal welfare groups appear to follow similar rationale to biologists and veterinarians in conjunction with organizational policies (e.g., RSPCA [62,63,64,65]). Veterinarians and veterinary bodies also hold certain special ethical obligations regarding animal welfare [67,68,112,113,114,115,122,128]. Some individual veterinarians have been strongly criticized for their involvement in reptile trading and keeping due to inherent harms (threats to animal welfare, public health and safety, species conservation, invasive alien organisms, antimicrobial resistance, and other issues), and related conflicts with obligations to ethical codes of conduct [136].

Guidance regarding enclosures for certain other vertebrates (e.g., dogs, cats, birds) recommends that dimensions provide for the ability to fully straighten (stretch) their bodies in any direction as absolute minimum conditions (e.g., Defra [118]). On this basis, the 1 × SL principle for snakes falls short of even the basic minimum standards imbedded in these other examples of guidance, because in most cases snakes would only be able to fully straighten in one linear dimension. Thus, an enclosure with only a single dimension allowing full stretching precludes the preference of snakes to straighten at will in any direction (particularly highlighted in the case of arboreal species). Accordingly, it is plainly implied that snakes ought to be able to fully straighten in all three dimensions, and therefore that proposals for a single maximum linear dimension accommodating the 1 x SL principle are incompatible with other accepted standards for animal health and welfare.

Some actors within the pet reptile trading and keeping sectors arguably have a strong vested interest in promoting enclosures <1 × SL; for example, motivations towards economy of holding spaces, whether at commercial or domestic premises [3], erroneous beliefs that greater space is simply unnecessary [3], and other misguided beliefs that smaller enclosures are otherwise beneficial to snakes (e.g., in particular by providing ‘greater security’, ‘avoiding agoraphobia’, and ‘promoting feeding and reproduction’ (see [5,8,39]). These examples provide several commonly recurring themes and justifications included throughout the trade and hobby literature for enclosures <1 × SL. However, some major pet industry guidance clearly states that all snakes should be able to stretch out and move freely within their enclosures (e.g., [129]).

Snake keepers and others with allied interests frequently interpret observations that individuals feed, grow, and reproduce ‘well’, and appear to be clinically healthy or ‘thriving’ as signs of good husbandry and animal welfare [5]. Nevertheless, as for other animals, snakes may manifest such signs that can apparently, but misleadingly, suggest good physical, physiological, and psychological health, while also simultaneously exhibiting established signs of captivity-induced stress (e.g., [5,6,22,27,28,95,137]).

### 4.4. Evidential Bases for Enclosures Allowing Other Animals the Ability to Fully Stretch

The English Government guidance for establishments selling pets (see below) allows as an absolute minimum for all animals (except snakes) the ability to fully stretch. It is reasonable to expect the same kinds of requirements for reptiles because there are no rational grounds to discriminate against snakes and their spatial needs. For example, regarding spatial needs for other animals, the English Government’s guidance [118] concerning pet selling establishments states the following:

For dogs, “The kennel area must be large enough to allow for separate sleeping and activity areas. The kennel must allow each dog to be able to walk, turn around and wag its tail without touching the sides of the kennel. The dogs must have sufficient room to play, stand on their hind limbs and to lie down without touching another individual. The kennel size required will increase in relation to the size and number of dogs housed at any one time. The length and width must be sufficient to allow all the dogs to lie outstretched without their noses or tails touching the walls or other individuals”.

For cats, this same source states “Cat units must be large enough to allow for separate sleeping and activity areas. The unit must allow each cat to be able to walk and turn around without touching the sides of the unit. The cats must have sufficient room to play, stand on their hind limbs and to lie down without touching another individual. The unit size required must increase in relation to the size and number of cats housed at any one time. The length and width must be sufficient to allow all the cats to lie outstretched without their noses or tails touching the walls or other individuals.”

For birds, “Where a bird uses a cage for sleeping, and the vast majority of the day is spent outside of the cage in a flight aviary where it is given the option to fly, then the cage must be a minimum of 1.5× the bird’s flying wingspan for each of the length, depth and height of the cage. For birds that spend the majority of their time in the cage, the cage must be a minimum of 2× the bird’s flying wingspan for the length, and 1.5× flying wingspan for the depth and height of the cage. A pair of birds must have enough space to fly past each other with the depth being increased to a minimum of 2× flying wingspan. In multiple occupancy cages, for every additional bird over two birds the cage dimensions must be increased by a set percentage per additional bird (either length or width or split between the two dimensions) of the individual’s flying wingspan for that.” In any event, legally binding provisions direct that all birds must be able to fully stretch (i.e., including wings) in all dimensions (e.g., [138]).

Thus, for dogs, cats, and birds, guidance requires provision of sufficient space in enclosures to enable all animals to fully stretch in all dimensions (and this minimum spatial principle is also been applied to reptiles other than snakes). This type of guidance is similarly stipulated in other formal resources (e.g., [139]). Relatedly, and importantly the English government (Defra) has confirmed (Freedom of Information Act response, Defra, 31.3.21) that no department holds scientific evidence to underpin its guidance that dogs, cats, and birds should be provided with enclosures in which as an absolute minimum they can fully stretch. Therefore, it may be presumed that the evidence base that has been accepted by the English Government for dogs, cats, and birds comprises information of primarily non-scientific anecdotal and opinion origin.

Accordingly, for existing recommendations allowing dogs, cats, and birds to fully stretch, the required scientific evidential threshold for these animals appears low. However, this approach is consistent with the accepted precautionary principle that where scientific evidence is lacking it is appropriate to err on the side of a better perceived animal welfare outcome [140]. Ironically, therefore, the evidence base (as firmly established by numerous scientific reviews and research publications) for snakes needing to fully stretch is significantly stronger than that for other animals already benefiting from greater than 1 × total body length enclosure provisions. Furthermore, hypothetically, should such evidence not exist, then the precautionary principle ought anyway to apply, thus demanding enclosures in which snakes can fully stretch in all dimensions as a default absolute minimum. Currently, many snakes are being denied this minimum space because accommodating their elongate morphology is inconvenient to some sellers or keepers, rather than any reason relating to their needs.

## 5. Conclusions and Recommendations

Snakes commonly adopt rectilinear—stretched-out—postures as part of their normal and essential locomotor and static behaviors, and this fact is uncontested. Expression of normal, often hard-wired, behavior such as locomotion, exploration, escape, rectilinear postures, or social interaction, as well as avoiding disturbances and conspecifics, is fundamental to animal welfare, and it is robustly evidenced in the literature. Smaller enclosures severely limit or prevent these essential actions. Snakes exhibit many recognized problematic behavioral and clinical conditions associated with restrictive enclosures and consequent confinement stress. While life in nature is not stress-free, there are clear obligations on caretakers to take all reasonable measures to minimize stress in animals under their management.

Critically examining the evidence base regarding recommendations for ≥1 × SL enclosure size reveals this information to be founded historically and currently on robust scientific research and associated rationales concerning snake behavior in the wild, home ranges, behavior in captivity, established principles of animal welfare science, negative clinical and behavioral consequences of imposed spatially restrictive environments, common practice, opinion, and avoidance of harm. All of this evidence and the informed objective opinion of experts, as well as all of the experimental research, support the assertion that absolute minimum spatial conditions for snakes must allow them enough room in which to fully stretch out, and that in smaller environments where snakes cannot stretch out, they suffer—even in temporary, short-term, conditions (e.g., those that persist beyond one circadian cycle/one day) [141,142]. In contrast, the evidence base regarding recommendations for <1 × SL reveal this information to lead back to a small number of literature items founded arbitrarily on economy of space, erroneous beliefs regarding snake biology, common traditional practice, and opinion. Furthermore, there is no evidence suggesting that snakes benefit from smaller, less enriched enclosures or that they are unharmed by such conditions. Although further work is welcome, recent studies (e.g., [8,26,31,35,37,38,41,143]) strongly cross-corroborate other works to confirm that snakes naturally occupy large home ranges, utilize available space, prefer more spacious and diverse habitats, and commonly adopt stretch-out postures, and that such postures are important for the avoidance of harm and achievement of quiescence and comfort.

The minimum 1 × SL recommendation for enclosures does not suggest that this provision offers adequate space for all necessary normal and important considerations, including exploration, suitable habitat, environmental diversity, thermal gradients, social interaction, exercise, roaming, and other factors. Rather, such enclosures are vital to allow for a limited number of essential behaviors, of which rectilinear posturing is one. Nevertheless, in practice, this recommendation means providing enclosures ≥1 × SL for a primary (straight line not diagonal) dimension as a default minimum condition, even for short-term housing (other than during brief transportation). Moreover, guidance for minimum ≥ 1 × SL enclosures is now mainstream objective advice within the scientific literature, and it is also being adopted by other evidence-based information users, such as pet insurers [144], DIY cage-build specifications [145], and the American pet industry [129].

Snake enclosures are commonly designed and furnished according to, for example, whether a species is characteristically terrestrial or arboreal, with arboreal species being afforded greater height dimensions (e.g., see [51]). However, many snake species exhibit regular behaviors outside of these norms and cross over between aquatic, semi-aquatic, fossorial, terrestrial, and arboreal habits. Therefore, rationally, the ≥1 × SL principle ought to be accommodated for all three primary (non-diagonal) dimensions, regardless of species habit, so that snakes can choose the direction in which they wish to stretch (e.g., in any horizontal or vertical dimensions). While our recommendations constitute scientific evidence-based guidance, it remains for formal authorities and other responsible interests to determine the manner in which such guidance may be implemented, for example, whether as part of relevant legal frameworks or other obligations, and any application of timescales.

Because some frameworks for recommendations can allow for margins of error (e.g., in cage manufacturing), these margins may potentially result in cages less than the full length of the snake; for example where a one-meter-long vivarium is marketed as suitable for a one-meter-long snake, and where the enclosure fails to meet that target. Thus, it is important for guidance to emphasize that enclosures must provide the ability for snakes to fully stretch. Table 7 provides summary highlight conclusions and recommendations regarding snake rectilinear behavior and enclosure requirements based on objective peer-reviewed evidence. Finally, in response to growth of the captive animal, enclosure size must be reviewed regularly as an essential requirement of responsible husbandry.

## Figures and Tables

**Table 1:** Broom’s [21] five factors illustrating where control, or lack of it, may impact animal welfare.

Factor	Description
1. Difficulties in movements	Environment features that restrict ability to move normally or adopt normal postures or positions.
2. Frustration	Animals knowing how to exercise controlled interactions with their environment, but being thwarted from performing them in a normal way.
3. Absence of specific input	Absence of essential stimuli.
4. Insufficient stimulation	Thwarted innate psychological and behavioral needs for stimuli in a low complexity environment—or sensory deprivation.
5. Overstimulation	Overload of stimuli.

**Table 2:** Mellor’s [25] Five Domains Model (reduced and summarized) illustrating positive states and animal welfare. Factors highlighted in bold text suggested as notably relevant to snake positive or negative welfare, and improved by greater space or hindered by lesser space.

Domain	Description
1. Nutrition	Opportunities to: **drink enough water; eat enough food; eat a balanced diet; eat a variety of foods; eating correct quantities.**
2. Environment	Available conditions: **thermally tolerable; suitable substrate; space for freer movement; fresh air; pleasant/tolerable odors; light intensity tolerable; noise exposure acceptable; normal environmental variability; predictability.**
3. Health	Little or no: **disease; injury; functional impairment;** **poisoning. Body condition appropriate; good fitness level.**
4. Behavior	‘Agency’ exercised via: **varied, novel, engaging environmental challenges; congenial sensory inputs; available engaging choices; free movement; exploration; foraging/hunting**; bonding/reaffirming bonds; rearing young; **playing; sexual activity; using refuges, retreat, or defensive attack; sleep/rest sufficient.**
5. Mental state	**Wetting/quenching pleasures of drinking**; **pleasures of different tastes/smells/textures;** pleasure of salt taste; masticatory pleasures; **postprandial satiety**; **gastrointestinal comfort; forms of comfort - thermal, physical, respiratory, olfactory,** auditory, **visual; variety-related comfort; comfort of good health and high functional capacity; vitality of fitness; calmness; engaged, in control;** affectionate sociability; maternally rewarded; **excitation/playfulness; sexual gratification; secure/protected/confident; likes novelty; energized/refreshed**.

**Table 3:** Summary of findings for peer-reviewed * information sources and recommendations consistent with or specifying ≥1 × snake length (SL) minimum enclosure size.

Reference	Information Source	Recommendation	Information/Evidence Base
Chiszar et al., 1995 [42]	Book chapter	Consistent with ≥1 × SL	Review/research
Gillingham, 1995 [11]	Book chapter	Consistent with ≥1 × SL	Review
Warwick, 1995 [28]	Book chapter	≥1 × SL	Review
Warwick and Steedman, 1995 [32]	Book chapter	Consistent with ≥1 × SL	Review
Divers, 1996 [43]	Journal article	Consistent with ≥1 × SL	Review
Astley and Jayne, 2007 [44]	Journal article	Consistent with ≥1 × SL	Research
Cannon and Johnson, 2011 [45]	Published proceedings	Consistent with ≥1 × SL	Review
Warwick et al., 2013 [34]	Journal article	≥1 × SL	Review
Barten and Fleming, 2014 [46]	Book chapter	Consistent with ≥1 × SL	Review
Jepson, 2015 ^†^ [47]	Journal article	≥1 × SL	Review
Wilkinson, 2015 [48]	Journal article	Consistent with ≥1 × SL	Review
Divers, 2018 [49]	Veterinary manual	Consistent with ≥1 × SL	Review
Warwick et al., 2018 [50]	Journal article	Consistent with ≥1 × SL	Review
Rossi, 2019 [51]	Book chapter	≥1 × SL	Review/research
Warwick et al., 2019 [8]	Journal article	≥1 × SL	Review
Cadenas and Martínez-Silvestre, 2020 [36]	Veterinary manual	Consistent with ≥1 × SL	Review
D’Cruze et al., 2020 [52]	Journal article	Consistent with ≥1 × SL	Review/research
Howell et al., 2020 [9]	Journal article	Consistent with ≥1 × SL	Review/research
Loughman, 2020 [37]	Journal article	Consistent with ≥1 × SL	Review/research
Spain et al., 2020 [38]	Journal article	Consistent with ≥1 × SL	Review/research
Hedley, 2020 [53]	Book chapter	≥1 × SL	Review
Kubiak, 2020 [54]	Book chapter	≥1 × SL	Review
Arena and Warwick (in press) [39]	Book chapter	≥1 × SL	Review
Warwick (in press) [27]	Book chapter	≥1 × SL	Review
Warwick and Steedman (in press) [40]	Book chapter	Consistent with ≥1 × SL	Review

* Article or chapter appearing in a peer-reviewed journal or book. Keys: ≥1 × SL = equal to or greater than total length of snake as minimum primary linear enclosure dimension. Consistent with ≥1 × SL recommendation (e.g., the snake must be provided with as much space as possible). ^†^ Jepson, 2015 allows for absolute minimum 1 × SL diagonal dimension. Note: Barten and Fleming, 2014 [46], and Wilkinson, 2015 [48], appear in both Table 3 and Table 4 because these sources provide recommendations for both <1 × SL and ≥1 × SL according to specific species.

**Table 4:** Summary of findings for peer-reviewed * information sources and recommendations consistent with or specifying <1 × snake length (SL) minimum enclosure size.

Reference	Information Source	Recommendation	Information/Evidence Base
Barnard, 1996 [55]	Book	75% SL	Review
De Vosjoli, 1999 [56]	Journal article	2/3 SL	Review
Griswold, 2001 [57]	Journal article	~50% SL	Review
Barten and Fleming, 2014 [46]	Book chapter	Length + width of cage = SL	Review
Wilkinson, 2015 [48]	Journal article	Consistent with <1 × SL	Review
Varga, 2019 [58]	Book chapter	Consistent with <1 × SL	Review

* Article or chapter appearing in a peer-reviewed journal or book. Keys: Consistent with <1 × SL recommendation as primary linear dimension of enclosure. Length + width of cage = SL = two dimensions combined equate to snake length. ~50% SL = approximately half snake length as minimum primary linear dimension of enclosure. 2/3 SL = two-thirds snake length as minimum primary linear dimension of enclosure. 75% SL = three-quarters snake length as minimum primary linear dimension of enclosure. Note: Barten and Fleming, 2014 [46], and Wilkinson, 2015 [48], appear in both Table 3 and Table 4 because these sources provide recommendations for both <1 × SL and >1 × SL according to specific species.

**Table 5:** Summary of findings from grey literature * for information sources and recommendations consistent with or specifying ≥ 1 × snake length (SL) minimum enclosure size.

Reference	Information Source	Author Credentials	Recommendation	Information/Evidence Base
Queensland Government, 1992 [110]	Government guidance	Anonymous	Small snakes Consistent with ≥1 × SL	Common practice, opinion
Barcelona, 2014 [111]	Government guidance	Anonymous	≥1 × SL	Common practice, consensus opinion
BVZS, 2014 [112]	Position paper	Veterinarians	≥1 × SL	Consensus
Arena et al., 2018 [113]	Report	Veterinarians, Biologists	≥1 × SL	Consensus
RSPCA, 2019 [62,63,64,65]	Online guidance	Biologist(s)	≥1 × SL	Consensus
Stidworthy and Doherty, 2019 [114]	Position declaration/published letter	Veterinarians	≥1 × SL	Consensus
BVA, 2020 [115]	Editorial	Veterinarian	Consistent with ≥1 × SL	Semi-scientific
Raynsford, 2020 [116]	Journal editorial	Veterinarian	≥1 × SL	Semi-scientific
RVC, 2021 [67]	Position paper/online guidance	Veterinarians	Consistent with ≥1 × SL	Consensus
RVC, 2021 [68]	Position paper/online guidance	Veterinarians	Consistent with ≥1 × SL	Consensus
Hollandt et al., submitted [26]	Journal article	Biologist(s)	≥1 × SL	Review/research

* Government or other report or scientific letter identified through Google Scholar. Keys: ≥1 × SL = equal to or greater than total length of snake as minimum primary linear enclosure dimension. Consistent with ≥1 × SL recommendation (e.g., must be provided with as much space as possible).

**Table 6:** Summary of findings from grey literature * for information sources and recommendations consistent with or specifying < 1 × snake length (SL) minimum enclosure size.

Reference	Information Source	Author Credentials	Recommendation	Information/Evidence Base
Basque Country, 2008 [117]	Government guidance	Anonymous	≥2/3 SL	Common practice, consensus opinion
NSW, 2013 [109]	Government guidance	Anonymous	0.5 SL	Common practice, consensus opinion
Kaplan, 2014 [108]	Online guidance	Biologist	Terrestrial/fossorial 75% SL	Common practice, opinion, ‘rule of thumb’
Barcelona, 2014 [111]	Government guidance	Anonymous	≥2/3 SL	Common practice, consensus opinion
Defra, 2018 [118]	Government guidance	Anonymous	≥2/3 SL	Common practice, opinion
Victoria State Government, 2020 [119]	Government guidance	Anonymous	0.45 SL	Common practice, opinion
Queensland Government, 2020 [110]	Government guidance	Anonymous	Larger snakes ~50% SL	Common practice, opinion

* Government or other report or scientific letter identified through Google Scholar. Keys: 0.45 SL = less than half snake length as minimum primary linear dimension of enclosure. 0.5 SL = half snake length as minimum primary linear dimension of enclosure. ~50% SL = approximately half snake length as minimum primary linear dimension of enclosure. >2/3 SL = equal to or greater than two-thirds snake length as minimum primary linear dimension of enclosure. 75% SL = three-quarters snake length as minimum primary linear dimension of enclosure.

**Table 7:** Summary highlight conclusions and recommendations regarding snake rectilinear (straight line/stretched out) behavior and enclosure requirements based on peer-reviewed evidence.

Rectilinear behavior is normal, distinct, and common across snake species.
2. Rectilinear behavior is essential and fundamental to snake health and welfare.
3. Snakes prefer larger and naturalistic environments, including in which they can fully stretch.
4. Snakes exhibit greater manifestations of behavioral, psychological, and clinical signs relating to stress and debilitation in enclosures in which they cannot fully stretch, both in short-term and long-term conditions.
5. No evidence found to suggest that snakes are unharmed by enclosures where they cannot fully stretch.
6. Evidence-base for recommendations <1 × SL is minimal and unscientific.
7. Evidence-base for recommendations >1 × SL is robust and scientific.
8. Scientific evidence for snakes needing to fully stretch in enclosures appears greater than that accepted for dogs, cats, and birds.
9. Objective scientific research and guidance determines that snakes must be able to fully stretch in all conditions, other than during, for example, essential brief transportation.
10. Scientific evidence-based recommendations for providing enclosures allowing snakes to fully stretch now constitute mainstream guidance information and good practice.
11. Snakes should be provided with environments that allow them to fully stretch their bodies in all three enclosure dimensions as a minimum, including in short-term situations.

## Data Availability

All data are included in the article.
